# The Performance and Metabolism of Dairy Cows Receiving an Ultra-Diluted Complex in the Diet during the Transition Period and Early Lactation

**DOI:** 10.3390/ani13203261

**Published:** 2023-10-19

**Authors:** Larissa S. Gheller, Mellory M. Martins, Thiago H. Silva, Gustavo Freu, Márcia S. V. Salles, Luiz C. R. Júnior, Weber V. B. Soares, Arlindo S. Netto

**Affiliations:** 1 Department of Animal Science, College of Animal Science and Food Engineering, University of São Paulo, Pirassununga 13635-900, SP, Brazil; larissa.gheller@usp.br (L.S.G.); mellory.martins@usp.br (M.M.M.); silvath@usp.br (T.H.S.); 2 Department of Animal Nutrition and Production, School of Veterinary Medicine and Animal Science, University of São Paulo, Pirassununga 13635-900, SP, Brazil; gustavofreu@usp.br; 3 Animal Science Institute (IZ), Ribeirão Preto 14030-640, SP, Brazil; marcia.saladini@gmail.com; 4 Animal Science Institute (IZ), Nova Odessa 13460-000, SP, Brazil; luiz.roma@sp.gov.br (L.C.R.J.); weber.soares@sp.gov.br (W.V.B.S.)

**Keywords:** homeopathy, liver health, periparturient, stress, udder health

## Abstract

**Simple Summary:**

The transition period is a critical phase for dairy cows, characterized by significant metabolic changes that occur to prepare the cow for calving and future lactation. This study evaluated the effects of an ultra-diluted complex on the productive performance and metabolic profile of dairy cows during the transition period and early lactation. Thirty multiparous pregnant cows were blocked and randomly assigned to either a placebo control (CON) group or an ultra-diluted complex (UD) group. Cows were evaluated from 30 days prior to calving through 60 days in milk. The addition of UD to the cows’ diet had no direct effect on the cows’ dry matter intake, but the cows in the UD treatment had a higher dry matter intake relative to their body weight. The group receiving UD showed a trend toward lower somatic cell counts, indicating better udder health. In addition, a trend toward better liver health was observed for cows in the UD group. These results suggest that the use of UD may be beneficial for dairy cows when used during the transition period and early lactation.

**Abstract:**

This study evaluated the effects of feeding an ultra-diluted complex to dairy cows during the transition period and early lactation. Thirty multiparous pregnant dairy cows were blocked and randomly assigned to either a placebo control (CON) group or ultra-diluted complex (UD) group. The CON group received a placebo (basal diet + 40 g/cow/day of expanded silicate), while the UD group received the ultra-diluted complex (basal diet + 40 g/cow/day of PeriParto Transição–RealH, composed of ultra-diluted substances + vehicle: expanded silicate). Cows were evaluated from 30 days before the expected calving date until 60 days in milk (DIM) for sample and data collection. Post-partum dry matter intake (DMI) was not affected by the treatment. Cows fed UD had higher DMI relative to BW. Feeding UD increased milk lactose content and decreased milk protein content. Cows fed UD had lower somatic cell counts in the third and fourth week of lactation. Cows fed UD showed a tendency for higher liver health index. Using UD during the transition period and early lactation may benefit liver and udder health of dairy cows with no detrimental effect on milk performance.

## 1. Introduction

Dairy cows’ transition period (TP) is the interval comprising 3 weeks before up to 3 weeks after calving [1]. TP is characterized by significant metabolic changes that occur to prepare the cow for calving and future lactation [2,3,4]. The transition from the dry period, a period of low metabolic demand, to lactation, a period of high nutrient demand for colostrum synthesis and increased milk production, can lead to metabolic imbalances with consequent impairment of immune function [5]. In addition, if cows are unable to adequately cope with the changes during this period, liver damage, systemic inflammation, and energy metabolism problems may be triggered [6]. These changes often result in an increased risk of health problems that can negatively affect the performance and well-being of dairy cows [6,7,8]. Ensuring a successful transition to lactation is extremely important to optimize the performance of dairy cows [9]. In this scenario, the adoption of nutritional strategies and management measures can be helpful in minimizing adverse effects on the metabolism of dairy cows undergoing the TP.

Several nutritional approaches have been studied to improve the transition of dairy cows from the dry period to the subsequent lactation [10,11]. Among these approaches, the use of compounds of natural origin [12,13,14] has received considerable attention, especially due to the global trend of reducing the use of antimicrobials in the livestock production system [15]. In this context, employing ultra-diluted compounds becomes an interesting and safe alternative to antibiotics [16]. The ultra-diluted compounds, also known as homeopathic compounds, were posited by Samuel Hahnemann in the 18th century, and are based on the principle “like cures like”, which suggests that substances that cause symptoms in healthy individuals can be used to treat similar symptoms in sick individuals [17,18]. The use of ultra-diluted compounds involves the use of highly diluted and dynamized substances to avoid toxicity and stimulate the body’s healing responses [17,19]. Even though the mechanisms of action behind the ultra-diluted compounds are not fully understood [20], previous research has already revealed that the use of ultra-diluted compounds in newborn calves was able to decrease the number of episodes of neonatal diarrhea [21], has the potential to reduce the occurrence of digestive disorders and reduce the number of days affected by tick-borne diseases in weaned calves, as well as minimize the risk of culling heifers [22]. On the other hand, other studies did not demonstrate the effect of the use of ultra-diluted compounds in the treatment of mastitis [23] and prevention of endometritis [24].

Despite significant scientific advances, studies with ultra-diluted compounds have shown highly controversial results [25]. Given the conflicting results and the limited number of studies with high scientific rigor using ultra-diluted compounds, there is clear evidence that we need to advance the frontiers of knowledge in this area. To our knowledge, no studies have evaluated the effects of ultra-diluted compounds in the diet of cows during TP. To fill this gap, the aim of this study was to investigate the effects of an ultra-diluted complex on the productive performance and metabolic profile of dairy cows during TP and early lactation. The ultra-diluted complex used in this study was developed for managing the stress-related issues and liver health of dairy cows during the TP, and consists of microminerals and homeopathic medications. According to Boericke [26], the compounds in the ultra-diluted complex used in our study can act by promoting cow productivity, providing preventive and curative benefits for enteritis and liver problems, as well as acting on calving ease, fertility problems, and post-traumatic recovery of cows. We hypothesized that cows receiving ultra-diluted complex in the diet have better performance and metabolic profile during TP and early lactation.

## 2. Materials and Methods

This study was conducted from June to October 2021 at the facilities of the Dairy Cattle Research Center, belonging to the São Paulo Agency for Agribusiness Technology, Animal Science Institute, located in Nova Odessa (São Paulo, Brazil). This study was approved by the Committee on Ethics in the Use of Animals of the Faculty of Animal Science and Food Engineering of the University of São Paulo, under number 651619012. Assuming a pooled standard deviation of 4 units, the study would require a sample size of 9 for each group (i.e., a total sample size of 18, assuming equal group sizes), to achieve a power of 80% and a level of significance of 5% (two sided), for detecting a true difference in means between the test and the reference group of 5.34 (i.e., 1.65–3.69) units, according to Zhou et al. [27].

### 2.1. Experimental Design, Treatments, and Diets

This study was a randomized, and placebo-controlled trial. Thirty crossbred (Holstein × Jersey) dairy cows, multiparous (3.7 ± 1.8 lactations, 545.6 ± 67.6 kg of body weight; BW, 2.60 ± 0.42 of body condition score; BCS), pregnant and expected to calve 30 days after the beginning of the evaluations were enrolled in this study. Cows were divided into blocks according to BCS, and parity. The cows were weighted using a walking-through scale (Toledo, MGR 3000 Campo, São Bernardo do Campo, Brazil). Body condition score determination was performed according to the method proposed by Edmonson et al. [28]. The cows were randomly assigned to the following treatments: (a) placebo control (CON, basal diet with the addition of vehicle only–expanded silicate 40 g/cow/day); and (b) UD (basal diet with the addition of 40 g/cow/day of the ultra-diluted complex (PeriParto Transição–RealH) composed by *Aletris acemose* 10^−12^ + *Aristolochia* 10^−14^ + *Arnica montana* 10^−14^ + *Arsenicum album* 10^−14^ + *Bellis perenis* 10^−14^ + *Berberis vulgaris* 10^−12^ + *Calcium carbonicum* 10^−30^ + *Carboneum tetrachloricum* 10^−30^ + *Cardus marianus* 10^−12^ + *Chelidonium majus* 10^−12^ + *China officinalis* 10^−12^ + *Chionantus virginica* 10^−30^ + *Cimicifuga racemosa* 10^−60^ + *Colibacilinum* 10^−18^ + *Colocynthis* 10^−18^ + *Croton tiglium* 10^−14^ + *Eberthinum* 10^−18^ + *Enterococinum* 10^−18^ + *Ferrum metallicum* 10^−18^ + *Gossypium* 10^−14^ + *Hypericum perforatum* 10^−60^ + *Ignatia amara* 10^−60^ + *Iodum* 10^−14^ + *Leptandra virginica* 10^−12^ + *Mercurius vivus* 10^−14^ + *Myrica cerifera* 10^−14^ + *Natrum muriaticum* 10^−60^ + *Oophorinum* 10^−12^ + *Paratyphoidinum* 10^−18^ + *Phosphorus* 10^−14^ + *Podophylinum peltaltum* 10^−30^ + *Pulsatilla* 10^−14^ + *Ruta graveolens* 10^−14^ + *Silicea terra* 10^−400^ + *Sulphur* 10^−18^ + *Symphytum officinale* 10^−60^ + *Tireoidinum* 10^−14^ + vehicle (expanded silicate, enough quantity for 1 kg)). The UD and the placebo were added during concentrate preparation to achieve an in-take of 40 g/cow/day according to the manufacturer’s instructions. The ultra-diluted complex used in this study was prepared according to the Materia Medica described by Boericke [26]. To achieve blinding of research and farm personnel, treatments were designated as A and B. Only the professor/advisor knew the treatment assignments and was not involved in treatment allocation and administration, field collection, or data analysis. Data were unblinded at the time of article writing.

Cows were evaluated from 30 days before expected calving until 60 days after calving. During the prepartum period (30 days before calving until the day of calving), cows were housed in collective paddocks with shaded areas. After calving, the cows were moved to a lactating cow paddock equipped with automatic feeding system where the cows were identified by electronic tags and had access to the feed (Intergado Ltd.a., Contagem, MG, Brazil). Throughout the experimental period, the cows were fed twice a day (7 a.m. and 2 p.m.) and had ad libitum access to water. Cows were also evaluated daily for ruminal acidosis signs (e.g., fecal changes, red limbs and hooves). 

The basal diets before and after calving were formulated according to NRC recommendations [29]. The diets offered to the cows were the same throughout the experimental period. Ingredients and chemical composition of the basal diet are presented in Table 1. Cows in both groups received the same basal diet except for the placebo or UD complex addition. Cows were fed CON or UD from 30 days pre-calving until 30 days post-calving. Sorghum silage was used as a unique roughage. Sorghum silage and concentrate were provided as a total mixed ration (TMR).

### 2.2. Dry Matter and Nutrient Intake 

During the prepartum period, the cows were housed in collective paddocks, which did not allow us to evaluate the individual dry matter intake (DMI) during this period. Thus, DMI was only evaluated during the postpartum period.

After calving, DMI was recorded daily using automatic feeders (Intergado Ltda, Contagem, MG, Brazil). Feed supply was adjusted to maintain a feeding rate target orts between 5 and 10% of the offered feed (on as-fed basis). Samples of sorghum silage and orts were collected daily throughout the experimental period. Weekly samples were pooled to form one sample per treatment per experimental week. Concentrate ingredient samples were collected whenever concentrate was prepared at the feed mill.

The feed and orts samples were pre-dried in a forced-air oven at 55 °C for 72 h and processed in a Wiley mill (MA340, Marconi, Piracicaba, Brazil) using a 1 mm sieve. These samples were then analyzed for dry matter (DM) (method 930.15; [31]), crude protein (CP) (N × 6.25; method 984.13; [31]), ether extract (EE) (method 920.39; [31]), ash (MM) (method 942.05; [31]) and neutral detergent fiber (NDF) using alpha amylase without the addition of sodium sulfite [32]. Non-fibrous carbohydrate (NFC) content was calculated according to Hall [30], where NFC (g/kg) = 1000 − [(CP + EE + MM + NDF)].

Feed samples were also analyzed for starch content using an enzymatic degradation method (Amyloglucosidase^®^, Novozymes, Curitiba, PR, Brazil) and absorbances were measured on a semi-automatic spectrophotometer (SBA-200, CELM^®^, São Caetano do Sul, SP, Brazil) according to Hendrix [33].

### 2.3. Milk Yield and Composition

Cows were milked twice daily (7 a.m. and 4 p.m.) and milk production was recorded electronically (Delpro^®^, DeLaval, Tumba, Sweden) during the whole experimental period. Milk samples proportional to the two milkings of the day were collected at weeks 1, 2, 4, 6, and 8 after calving. The samples were stored in tubes containing 2-bromo-2-nitropropane-1-3-diol, homogenized, and sent to the laboratory for analysis. Concentrations of fat, protein, and lactose were determined by infrared absorption (Bentley 2000^®^). In addition, somatic cell count (SCC) was analyzed by flow cytometry using the Somacount 300^®^ (Bentley Instruments Inc., Chasca, MN, USA). 

Milk yield was corrected for 3.5% fat (FCM) according to described by Sklan et al. [34].

### 2.4. Colostrum Yield and Quality

All cows were followed through calving and the first milking was performed within two hours of calving. The quantity and quality of colostrum produced were recorded. Colostrum quality was measured using an optical Brix refractometer (Sper Scientific 300001 Refractometer, Brix 0–32%). Briefly, approximately 50 μL of colostrum was placed on the prism of the optical refractometer, where Brix readings were taken. Estimation of immunoglobulin concentration was performed by exposing the refractometer to a point of light that allowed the identification of a blue line on the scale, dividing it into light and dark colors [35]. All readings were performed by the same evaluator.

### 2.5. Blood Metabolites

Blood samples (~10 mL) were collected on days 30 (actual: 29.3 ± 2.96) and 7 (actual: 8.9 ± 2.85) before the expected calving date, on the calving day (d0), and on days 7 and 30 after calving. Blood samples were collected by puncture of the jugular vein using a vacutainer, always before the morning feed. Vacuolated tubes with clot activator to obtain serum and tubes with sodium fluoride to obtain plasma were used.

Immediately after sampling, the samples were centrifuged at 2000× *g* for 15 min, and the serum and plasma were kept frozen until further analysis. Glucose (plasma samples), cholesterol, urea, liver enzymes [aspartate aminotransferase (AST) and gamma-glutamyltransferasase (GGT)], bilirubin, calcium, and phosphorus concentrations were analyzed in serum samples using commercial biochemistry kits according to the manufacturer’s instructions (glucose: Ref. 133-1/500; total cholesterol: Ref. 76-2/100; urea: Ref. 104-4; AST: Ref. 109-4/30; and GGT: Ref. 105-2/30; total bilirubin: Ref. 94-1/104; total calcium Ref. 90-2/60; phosphorus Ref. 12-200; Labtest, Lagoa Santa, Brazil). The measurements were performed in an automatic biochemical analyzer (Mindray, BS 120, Shenzhen, China).

Acute phase proteins (Immunoglobulin A, ceruloplasmin, transferrin, albumin, haptoglobin, and immunoglobulin G) were measured in serum samples. Serum protein was determined by the biuret method using a commercial kit (Total Protein Ref. 99-100; Labtest, Lagoa Santa, Brazil). The separation of protein fractions was performed by acrylamide gel electrophoresis with sodium dodecyl sulfate (SDSPAGE), according to Laemmli [36]. After fractionation, the gel was stained with Coomassie blue solution (50.0% methanol, 40.0% water, 9.75% glacial acetic acid, and 0.25% Coomassie blue) for 10 min. The gel was then placed in a 7.0% acetic acid solution to remove excess dye until the protein fractions were clear. Acute phase protein concentrations were determined using a computerized densitometer.

Plasma concentrations of non-esterified fatty acids (NEFA) and beta-hydroxybutyrate (BHB) were measured in plasma samples using enzymatic kits (RANDOX Laboratories-Life Sciences Ltd. Crumlin, UK; BHB: Ranbut-Ref. RB1007; AGNE: Nefa Ref. FA115). The measurements were determined using an automatic system for biochemistry (SBA 200, CELM, Barueri, SP, Brazil).

The liver health index (LHI) was also calculated to characterize the liver function and inflammatory status of the cows. The LHI was calculated according to Kerwin [37], using the individual concentrations of albumin, cholesterol and bilirubin and the mean and standard deviation of the collected population, according to the following equation:

$$\mathrm{LHI}=\left[\left(\mathrm{Albumin}-\mu_{\mathrm{Albumin}}\right)/\sigma_{\mathrm{Albumin}}\right]+\left[\left(\mathrm{Cholesterol}-{\;\mu }_{\mathrm{Cholesterol}}/\sigma_{\mathrm{Cholesterol}}\;\right)\right]\;-\left[\left(\mathrm{Bilirrubin}-{\;\mu }_{\mathrm{Bilirrubin}}/\sigma_{\mathrm{Bilirrubin}}\;\right)\right]\;$$

where µ is the overall sampling population mean and σ is the overall sampling population standard deviation.

### 2.6. Statistical Analysis

Data were analyzed with SAS (version 9.4, Statistical Analysis System Institute Inc., Cary, NC, USA). Normality of residuals and homogeneity of variances were checked using PROC UNIVARIATE. Six cows were removed from the study due to twin calving (*n* = 1; 1 from CON group and 0 from UD group) or involuntary culling (*n* = 5; 2 from CON group and 3 from UD group), leaving data from 24 cows (12 from CON group and 12 from UD group) for statistical analysis.

Data of dry matter and nutrient intake, milk production and composition, BW and blood variables, were analyzed as repeated measures over time using PROC MIXED according to the following model:

$$Y_{ijk}=µ+B_{i}+T_{j}+D_{k}+{\left(T\times D\right)}_{jk}+e_{ijk},$$

where: *Y_ijk_* is the dependent variable; µ is the overall mean; *B_i_* is the block random effect; *T_j_* is the treatment fixed effect; *D_k_* is the sampling day fixed effect; (*T* × *D*)*_jk_* is the interaction between treatment and sampling day; and *e_ijk_* is the residual error. A first-order autoregressive covariance structure [AR (1)] was used for the dry matter and nutrient intake, and milk yield and composition data. This covariance structure is suitable for data collected at equal intervals and assumes decreasing correlations as a function of time. For BW and blood variables, the spatial power covariance matrix [sp(pow)] was used, which is specific for data collected at different time intervals. Weight at the beginning of the experiment was used as a covariate, tested for all variables, and kept in the model when *p* ≤ 0.05. Data on the blood sample collected on day −30 (actual: 29.3 ± 2.96) relative to the calving date was used as a covariate and forced into the models. 

The SCC was log-transformed according to Schukken et al. [38] to attend the data normal distribution:

CCS=Log2 (CCS100)+3.

Colostrum-related data were analyzed using PROC MIXED according to the following model:

$$Y_{ij}=µ+B_{i}+T_{j}+e_{ij}$$

where: *Y_ij_* is the dependent variable; µ is the overall mean; *B_i_* is the block random effect; *T_j_* is the treatment fixed effect; and *e_ij_* is the residual error.

Values are presented as least squares means. All analyses were considered significant if *p* ≤ 0.05 and tendency if *p* > 0.05 and ≤0.10.

## 3. Results

During the study period, four cows were treated for clinical mastitis (2 from CON and 2 from UD), two cows were treated for retained placenta (1 from CON and 1 from UD) and one cow (from UD group) was treated for hypocalcemia. None of the cows showed any signs of ruminal acidosis during the study period.

Cows included in this study were 3.7 ± 1.8 lactations, 545.6 ± 67.6 kg BW and 2.60 ± 0.42 ECC at the beginning of the evaluations. No differences in cows’ weight were observed between treatments (*p* = 0.459). Cows fed UD had higher DMI relative to body weight (Table 2; *p* = 0.034) during early lactation, especially in the first 4 weeks (*p* = 0.049). The CP intake tended to be higher (*p* = 0.064) for cows receiving UD during the TP, especially between weeks 5 and 8 of lactation (*p* = 0.053). Dry matter, organic matter, neutral detergent fiber, and ether extract intake were not affected (*p* ≥ 0.186) by treatment. No interactions between treatment and time were found for the variables evaluated (*p* ≥ 0.119).

**Figure 1:**
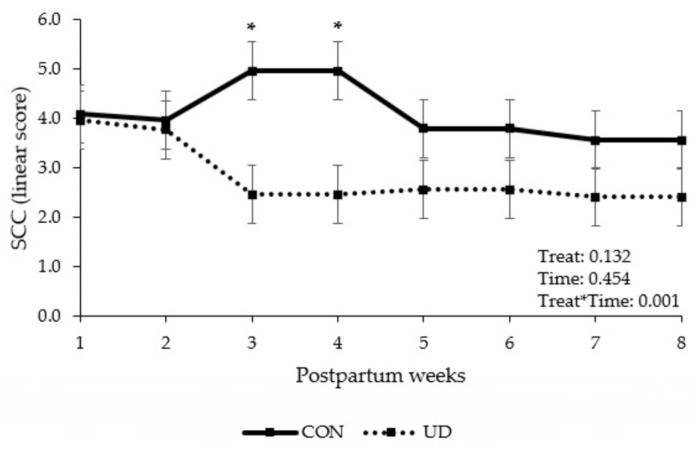
Somatic cell count (SCC) linear score of dairy cows in control or ultra-diluted complex fed during transition period and early lactation. CON: cows received placebo treatment (expanded silicate, 40 g/cow/day); UD: cows received ultra-diluted complex (PeriParto Transição–RealH, 40 g/cow/day). Error bars are SEM (standard error of the mean). Asterisks represent differences between CON and UD where *p* ≤ 0.05.

Feeding UD increased (Table 3; *p* = 0.024) milk lactose content (%) between 5 and 8 weeks after calving. There was an interaction between treatment and time (*p* = 0.0001) for milk protein content, with cows fed UD having lower milk protein content, especially during the first 4 weeks of lactation. An interaction between treatment and time (Figure 1; *p* = 0.001) was also found for SCC, with cows fed UD having lower SCC at 3 and 4 weeks post calving. No treatment effect (*p* ≥ 0.119) or interaction between time and treatment (*p* ≥ 0.138) were found for milk yield, FCM, fat, protein and lactose yield, milk fat content and the ratio between DMI and milk yield and between DMI and FCM. The quantity and quality of colostrum produced did not differ (*p* ≥ 0.102) between CON and UD group cows.

**Figure 2:**
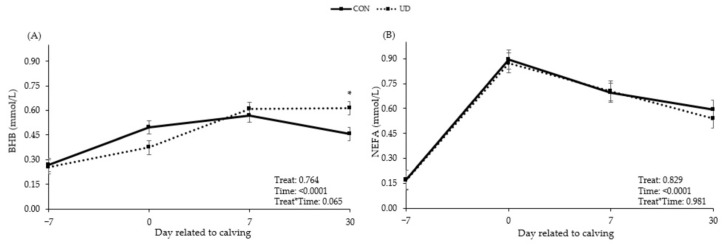
Plasma concentrations of beta-hydroxybutyrate (BHB; (**A**)) and non-esterified fatty acids (NEFA; (**B**)) of dairy cows in control or ultra-diluted complex fed during transition period and early lactation. CON: cows received placebo treatment (expanded silicate, 40 g/cow/day); UD: cows received ultra-diluted complex (PeriParto Transição–RealH, 40 g/cow/day). Error bars are SEM (standard error of the mean). Asterisk represents statistical tendency between CON and UD where *p* > 0.05 and ≤ 0.10.

**Figure 3:**
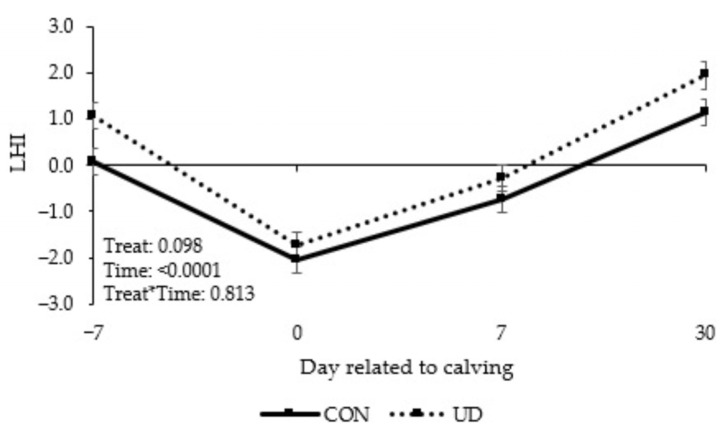
Liver health index (LHI) of dairy cows in control or ultra-diluted complex fed during transition period and early lactation. CON: cows received placebo treatment (expanded silicate, 40 g/cow/day); UD: cows received ultra-diluted complex (PeriParto Transição–RealH, 40 g/cow/day). Error bars are SEM (standard error of the mean).

There was a tendency for interaction between time and treatment (Figure 2; *p* = 0.065) for plasma BHB concentration, with cows fed UD tending to have higher BHB concentrations on day 30 after calving. On the other hand, no treatment effects or interactions between treatment and time were observed for NEFA (Figure 2; *p* ≥ 0.829), biochemical profile variables (Table 4; *p* ≥ 0.247), and acute phase proteins (Table 5; *p* ≥ 0.213). Cows fed UD tended to have a higher LHI (Figure 3; *p* = 0.098).

## 4. Discussion

This study provides the first scientific insights into the use of UD in the diet of transition and early lactation cows. Cows receiving UD tended to have a higher LHI than cows in the CON group. Higher post-calving LHI scores have been reported with better performance of cows throughout lactation, which is associated with reduced disease incidence, increased milk production, and a higher probability of pregnancy in the first 150 days in milk (DIM) [37]. Studies evaluating liver function index by albumin, cholesterol, and retinol concentrations reported lower milk production in early lactation in cows with impaired liver function [27,39]. However, although cows in the CON group had a lower LHI in our study, milk yield did not differ significantly between treatments.

Feeding UD resulted in a tendency to higher plasma levels of BHB at 30 days postpartum. Ketone elevation after calving is a normal adaptive response, but accumulation is not [40]. Hyperketonemia in dairy cows is defined when the plasma BHB concentration is equal to or greater than 1.2 mmol/L [41,42,43,44]. In addition, a cut-off point of 0.62 mmol/L for hyperketonemia has already been considered [45]. Mahrt et al. [46], who evaluated the prevalence of hyperketonemia in dairy cows up to 42 days of lactation, found an average prevalence of 11.8% (ranging from 9.6% during the first two weeks of lactation to 14.6% between the fifth and sixth weeks of lactation). Even considering the cut-off point given by Fürll [45], where our cows would be at the upper limit considered, we concluded that the cows in our study did not show any degree of hyperketonemia, as the highest BHB level found in this study was 0.610 mmol/L. Although cows may be at risk for hyperketonemia up to the sixth week of lactation [46], we believe that the BHB tendency in our study may have occurred by chance, as BHB levels are quite sensitive and may vary for several reasons, including daily variations in body fluids [47].

Cows fed UD during transition and early lactation had higher ratio between DMI and BW, especially during the first 4 weeks of lactation. Assuming that the cows on the UD treatment had better metabolic conditions during early lactation due to better LHI, this may have resulted in an increased DMI relative to BW [48,49]. Although we found no significant effects related to fatty acids, cows in the CON group may have suffered some degree of hepatic fatty acid oxidation, which is directly related to decreased appetite and inhibition of feeding behavior [50]. Also, cows receiving UD treatment tended to have higher CP intake, which may be considered a reflection of the increase in DMI relative to BW.

Regarding milk yield and components, lactose and protein content were affected by treatment. Milk produced by cows fed UD had higher lactose content, especially between weeks 5 and 8 of lactation, while milk from UD-fed cows had lower protein content, especially during the first 4 weeks of lactation. The lactose content of cow’s milk can be influenced by udder health [51], metabolism, and energy balance [52]. Although we did not find a significant effect on milk yield, higher lactose content may result in increased milk yield because lactose acts as an osmotic component in milk, stimulating secretion of water into milk via the bloodstream [53]. Also, because cows fed UD had higher DM:BW, they may have had higher ruminal production of propionate and consequently more precursors for gluconeogenesis [54], since glucose is the main precursor for lactose synthesis in the mammary gland [55]. The lower protein content in milk can be explained by the dilution effect, because although we found no significant effect on milk yield, cows from the UD treatment produced numerically more milk than cows from the CON treatment.

Cows receiving the UD treatment had lower SCC than cows in the CON group, especially between weeks 3 and 4 after calving. SCC in milk has been used as an indicator of udder health and immune status [56,57]. Since UD treated cows had a better LHI, we can speculate that these cows also had a better immune response in early lactation. Improving the cow’s immune system have been associated with reduced SCC in milk [58,59]. Additionally, high SCC has been reported by changing the milk composition [51], which could also explain the differences in lactose and protein content found in our study. However, changes in milk composition are dependent on the type of pathogen causing mastitis [50], but this assessment was beyond the scope of our study. The relations between UD complex and cows’ immune system need to be further investigated. 

Our study focused on presenting results related to performance and metabolism of dairy cows receiving UD during the TP, but has some limitations. The UD used in this study is composed of many ultra-diluted substances, making it difficult to identify isolated effects of each component. Due to the small number of cows involved in this study, the results should be viewed and interpreted with caution as this may have reduced the statistical power to detect differences between treatments. Future larger and more detailed studies evaluating the use of UD in the diets of transition and early lactation cows could use this work as a starting point. In addition, although our results did not improve the understanding of the mechanisms of action behind the use of UD, they will contribute to the scientific community; to our knowledge this is the first study evaluating the use of UDs during TP and early lactation of dairy cows.

## 5. Conclusions

UD use during the TP and early lactation did not affect cow production. However, the use of UD may have beneficial effects when it comes to udder and liver health. Our results offer fresh perspectives on the impact of UD use on the metabolism and performance of dairy cows during early lactation. Our findings can serve as a great starting point for future research evaluating the potential use of ultra-diluted complexes during the TP. 

## Figures and Tables

**Table 1:** Ingredients and chemical composition of experimental diets.

Experimental Diets		
*Ingredient* (g/kg DM)	Prepartum	Postpartum
Sorghum silage ^1,2^	716	600
Ground corn	112	219
Soybean meal 48% CP	112	156
Urea	10.7	4.50
Bicalcium phosphate	-	4.20
Limestone	-	3.60
Mineral premix ^3,4^	45.1	8.20
Salt	-	2.70
Ultra-diluted complex ^5,6^	4.20	1.80
*Chemical composition* ^7,8^ (g/kg DM)		
Dry matter, g/kg as fed	333	403
Ash	75.0	66.0
Organic matter	925	934
Crude protein	125	172
Ether extract	24.7	26.1
Neutral detergent fiber	528	413
Acid detergent fiber	375	276
Starch	166	238
Non-fiber carbohydrate ^9^	290	361
Total digestible nutrients ^10^	592	616
Lignin	62.4	47.4
NE_L_, Mcal/kg ^10^	1.33	1.49

^1^ Prepartum silage chemical composition (DM basis): 303 g/kg as-fed, 87 g/kg CP, 594 g/kg NDF, and 150 g/kg starch. ^2^ Postpartum silage chemical composition (DM basis): 300 g/kg as-fed, 86.0 g/kg CP, 593 g/kg NDF, and 159 g/kg starch. ^3^ Each kg of prepartum contained: 35 IU Biotin, 260 g Ca, 40 g Cl, 15 mg Co, 505 mg Cu, 15 mg Cr, 110 g S, 200 mg F, 20 g P, 30 mg I, 10 g Mg, 1500 mg Mn, 15 mg Se, 25 g Na, 2015 mg Zn, 200,000 IU Vitamin A, 20,000 IU Vitamin D3, 725 IU Vitamin E, 500 mg sodium monensin. ^4^ Each kg of postpartum contained: 240 g Ca, 30 g Co, 1010 mg Cu, 80 g S, 400 mg F, 60 mg I, 20 g Mg, 3000 mg Mn, 30 mg Se, 60 g Na, 4030 mg Zn, 400,000 IU Vitamin A, 40,000 IU Vitamin D3, 1450 IU Vitamin E, 1100 mg sodium monensin. ^5^ Placebo control (CON) diet, addition of vehicle (expanded silicate). ^6^ Ultra-diluted complex (UD) diet, addition of ultra-diluted complex composed of *Aletris acemose* 10^−12^ + *Aristolochia* 10^−14^ + *Arnica montana* 10^−14^ + *Arsenicum album* 10^−14^ + *Bellis perenis* 10^−14^ + *Berberis vulgaris* 10^−12^ + *Calcium carbonicum* 10^−30^ + *Carboneum tetrachloricum* 10^−30^ + *Cardus marianus* 10^−12^ + *Chelidonium majus* 10^−12^ + *China officinalis* 10^−12^ + *Chionantus virginica* 10^−30^ + *Cimicifuga racemosa* 10^−60^ + *Colibacilinum* 10^−18^ + *Colocynthis* 10^−18^ + *Croton tiglium* 10^−14^ + *Eberthinum* 10^−18^ + *Enterococinum* 10^−18^ + *Ferrum metallicum* 10^−18^ + *Gossypium* 10^−14^ + *Hypericum perforatum* 10^−60^ + *Ignatia amara* 10^−60^ + *Iodum* 10^−14^ + *Leptandra virginica* 10^−12^ + *Mercurius vivus* 10^−14^ + *Myrica cerifera* 10^−14^ + *Natrum muriaticum* 10^−60^ + *Oophorinum* 10^−12^ + *Paratyphoidinum* 10^−18^ + *Phosphorus* 10^−14^ + *Podophylinum peltaltum* 10^−30^ + *Pulsatilla* 10^−14^ + *Ruta graveolens* 10^−14^ + *Silicea terra* 10^−400^ + *Sulphur* 10^−18^ + *Symphytum officinale* 10^−60^ + *Tireoidinum* 10^−14^ + vehicle (expanded silicate, enough quantity for 1 kg). ^7^ Estimated by NRC [29], for prepartum cows, 550 kg body weight, eating 3 kg of concentrate per day. ^8^ Estimated by NRC [29], for fresh cows, 550 kg body weight, 3.5% fat in milk, 30 kg/d of milk yield and 2.98% true protein in milk, eating 10kg of concentrate per day. ^9^ Estimated according to Hall [30]. ^10^ Estimated according to NRC [29].

**Table 2:** Nutrient intake and body weight of dairy cows in control or ultra-diluted complex fed during transition and early lactation.

Variable	Treatments ^1^		SEM ^2^	*p*-Value		
	CON	UD		Treat	Time	Treat × Time
Postpartum intake, kg/d						
Dry matter						
1–60 d	18.2	19.9	0.79	0.186	<0.0001	0.433
Week 1–4	16.3	16.7	0.86	0.755	0.001	0.454
Week 5–8	20.7	22.5	0.90	0.198	0.183	0.378
Dry matter, % BW ^3^						
1–60 d	3.53	3.94	0.13	0.034	<0.0001	0.197
Week 1–4	2.94	3.40	0.15	0.049	<0.0001	0.119
Week 5–8	4.13	4.46	0.17	0.149	0.082	0.320
Organic matter						
1–60 d	17.0	18.6	0.74	0.194	<0.0001	0.434
Week 1–4	15.2	15.6	0.80	0.761	0.001	0.454
Week 5–8	19.4	21.0	0.84	0.208	0.184	0.375
Crude protein						
1–60 d	3.13	3.54	0.13	0.064	<0.0001	0.400
Week 1–4	2.90	3.03	0.15	0.569	0.001	0.440
Week 5–8	3.48	3.91	0.14	0.053	0.282	0.302
Neutral detergent fiber						
1–60 d	7.81	7.70	0.37	0.828	<0.0001	0.393
Week 1–4	6.51	6.57	0.41	0.918	<0.0001	0.249
Week 5–8	8.70	9.19	0.39	0.406	0.086	0.375
Ether extract						
1–60 d	0.47	0.52	0.02	0.195	<0.0001	0.345
Week 1–4	0.43	0.44	0.02	0.825	0.002	0.396
Week 5–8	0.54	0.58	0.02	0.182	0.172	0.472
BW ^4^	521.73	510.06	19.97	0.459	<0.0001	0.792

^1^ CON: cows received placebo treatment (expanded silicate, 40 g/cow/day); UD: cows received ultra-diluted complex (PeriParto Transição–RealH, 40 g/cow/day). ^2^ Standard error of the mean. ^3^ Ratio between DMI and body weight. ^4^ Body weight. The measurements were performed on days 30 (actual: 29.3 ± 2.96) and 7 (actual: 8.9 ± 2.85) before the expected calving date, on the calving day (d0), and on days 7 and 30 after calving.

**Table 3:** Milk yield and composition of dairy cows in control or ultra-diluted complex fed during transition and early lactation.

Variable	Treatments ^1^		SEM ^2^	*p*-Value		
	CON	UD		Treat	Time	Treat × Time
Milk, kg/d						
1–60 d	28.4	29.2	1.39	0.428	<0.0001	0.646
Week 1–4	27.9	29.0	1.42	0.309	<0.0001	0.738
Week 5–8	28.9	29.8	1.38	0.410	0.119	0.204
3.5% FCM, kg/d ^3^						
1–60 d	30.3	29.4	1.37	0.592	0.008	0.874
Week 1–4	30.3	29.2	1.43	0.523	0.019	0.896
Week 5–8	30.2	29.6	1.43	0.714	0.733	0.693
Fat, kg/d						
1–60 d	1.11	1.04	0.053	0.365	0.390	0.765
Week 1–4	1.13	1.03	0.062	0.289	0.256	0.740
Week 5–8	1.09	1.05	0.054	0.575	0.874	0.895
Protein, kg/d						
1–60 d	0.88	0.88	0.040	0.934	<0.0001	0.254
Week 1–4	0.92	0.92	0.041	0.832	0.0001	0.341
Week 5–8	0.85	0.84	0.044	0.700	0.955	0.537
Lactose, kg/d						
1–60 d	1.24	1.30	0.064	0.238	<0.0001	0.898
Week 1–4	1.21	1.28	0.070	0.187	<0.0001	0.748
Week 5–8	1.28	1.32	0.069	0.428	0.233	0.541
Fat, %						
1–60 d	3.90	3.63	0.146	0.119	0.800	0.719
Week 1–4	4.02	3.62	0.209	0.128	0.491	0.537
Week 5–8	3.80	3.64	0.145	0.382	0.966	0.968
Protein, %						
1–60 d	3.14	3.03	0.070	0.313	<0.0001	0.0001
Week 1–4	3.30	3.21	0.072	0.371	<0.0001	0.002
Week 5–8	2.97	2.86	0.069	0.301	0.060	0.076
Lactose, %						
1–60 d	4.40	4.51	0.039	0.055	<0.0001	0.138
Week 1–4	4.36	4.45	0.044	0.183	<0.0001	0.145
Week 5–8	4.42	4.57	0.041	0.024	0.755	0.311
Efficiency, Milk:DMI ^4^	1.63	1.60	0.065	0.749	<0.0001	0.409
Efficiency, FCM:DMI ^5^	1.69	1.63	0.071	0.551	<0.0001	0.458
Colostrum						
Yield, kg	4.25	5.63	0.66	0.102	-	-
Quality, °brix	25.6	27.7	1.20	0.232	-	-

^1^ CON: cows received placebo treatment (expanded silicate, 40 g/cow/day); UD: cows received ultra-diluted complex (PeriParto Transição–RealH, 40 g/cow/day). ^2^ Standard error of the mean. ^3^ Fat corrected milk at 3.5%, calculated according to Sklan et al. [32]. ^4^ Milk yield to dry matter intake ratio. ^5^ 3.5% Fat-corrected milk to dry matter intake ratio.

**Table 4:** Blood biochemical profile of dairy cows fed control or ultra-diluted complex during transition and early lactation.

Variable ^1^	Treatments ^2^		SEM ^3^	*p*-Value		
	CON	UD		Treat	Time	Treat × Time
AST (U/L) ^4^	75.45	73.72	2.345	0.590	<0.0001	0.743
Bilirubin (µmol/L)	2.389	2.295	0.227	0.394	<0.0001	0.916
Calcium (mg/dL)	9.304	9.563	0.159	0.257	<0.0001	0.953
Cholesterol (mg/dL)	72.79	79.71	4.085	0.247	<0.0001	0.375
Phosphorus (mg/dL)	5.396	5.582	0.148	0.382	0.0009	0.292
GGT (U/L) ^5^	22.63	23.44	0.572	0.334	0.050	0.536
Glucose (mg/dL)	67.19	69.89	3.956	0.474	<0.0001	0.700
Total protein (g/dL)	6.692	6.815	0.098	0.388	<0.0001	0.402
Urea (mg/dL)	28.60	27.28	1.195	0.445	<0.0001	0.674

^1^ Blood samples were collected on days −30 (covariate), −7, 0, +7, and +30 days relative to calving. ^2^ CON: cows received placebo treatment (expanded silicate, 40 g/cow/day); UD: cows received ultra-diluted complex (PeriParto Transição–RealH, 40 g/cow/day). ^3^ Standard error of the mean. ^4^ Aspartate aminotransferase. ^5^ Gama glutamil transferase.

**Table 5:** Acute phase proteins blood concentrations of dairy cows fed control or ultra-diluted complex during the transition period and early lactation.

Variable ^1^	Treatments ^2^		SEM ^3^	*p*-Value		
	CON	UD		Treat	Time	Treat × Time
Albumin (g/L)	49.93	44.60	0.554	0.413	<0.0001	0.673
Ceruloplasmin (mg/dL)	50.13	54.81	4.748	0.358	0.036	0.808
Haptoglobin (mg/dL)	48.09	51.11	4.179	0.618	<0.0001	0.363
IgA (mg/dL)	71.20	71.79	2.393	0.835	<0.0001	0.213
Transferin (mg/dL)	184.44	188.44	7.854	0.748	0.1186	0.901
IgG (mg/dL)	1495.01	1575.73	63.19	0.377	<0.0001	0.395

^1^ Blood samples were collected on days −30 (covariate), −7, 0, +7, and +30 days relative to calving. ^2^ CON: cows received placebo treatment (expanded silicate, 40 g/cow/day); UD: cows received ultra-diluted complex (PeriParto Transição–RealH, 40 g/cow/day). ^3^ Standard error of the mean.

## Data Availability

The raw data of this study will be made available by the authors (corresponding author) to any qualified researcher.
